# Ubiquitin-specific protease 22 is a deubiquitinase of CCNB1

**DOI:** 10.1038/celldisc.2015.28

**Published:** 2015-10-13

**Authors:** Zhenghong Lin, Can Tan, Quan Qiu, Sinyi Kong, Heeyoung Yang, Fang Zhao, Zhaojian Liu, Jinping Li, Qingfei Kong, Beixue Gao, Terry Barrett, Guang-Yu Yang, Jianing Zhang, Deyu Fang

**Affiliations:** 1 Department of Pathology, Northwestern University Feinberg School of Medicine, Chicago, IL, USA; 2 Department of Medicine, Northwestern University Feinberg School of Medicine, Chicago, IL, USA; 3 School of Life Science and Medicine, Dalian University of Technology, Panjin, China

**Keywords:** USP22, CCNB1, APC8, cell cycle, tumorigenesis

## Abstract

The elevated level of CCNB1 indicates more aggressive cancer and poor prognosis. However, the factors that cause CCNB1 upregulation remain enigmatic. Herein, we identify USP22 as a CCNB1 interactor and discover that both USP22 and CCNB1 are dramatically elevated with a strong positive correlation in colon cancer tissues. USP22 stabilizes CCNB1 by antagonizing proteasome-mediated degradation in a cell cycle-specific manner. Phosphorylation of USP22 by CDK1 enhances its activity in deubiquitinating CCNB1. The ubiquitin ligase anaphase-promoting complex (APC/C) targets USP22 for degradation by using the substrate adapter CDC20 during cell exit from M phase, presumably allowing CCNB1 degradation. Finally, we discover that USP22 knockdown leads to slower cell growth and reduced tumor size. Our study demonstrates that USP22 is a CCNB1 deubiquitinase, suggesting that targeting USP22 might be an effective approach to treat cancers with elevated CCNB1 expression.

## Introduction

Colorectal cancer is the second leading cancer killer in the United States [1]. Despite successful surgical removal of colorectal tumors, a significant fraction of patients show a recurrence of the disease and metastasis. New drug discovery has encountered difficulty due to lack of appropriate target gene(s) and related gene function studies. Recently, 11 genes have been identified as having death-from-cancer signatures displaying stem cell-like gene expression profiles in diverse solid tumors characterized by high malignancy and metastatic dissemination [2, 3]. One of the genes is *cyclin B1* (*CCNB1*), a member of the cyclin family, is a regulatory subunit of cyclin-dependent kinase 1 (CDK1) that begins to increase in the cytoplasm through the G2 phase, translocates to the nucleus during prophase, peaks in mitosis, and is rapidly degraded before the cell cycle is completed [4, 5]. Cyclin B1 is particularly critical for the maintenance of the mitotic state [6]. The degradation of CCNB1 is mediated by the E3 ubiquitin ligase, anaphase-promoting complex/cyclosome (APC/C) [5]. Because it is an essential cell cycle regulator, malfunctioning of CCNB1 might be proto-oncogenic, as its expression is often upregulated in various types of human cancers, including colon cancer [7–9], cervical cancer [10] and renal cancer [11]. Overexpression of CCNB1 leads to unscheduled cell cycle entry, uncontrolled cell proliferation and tumorigenesis [9, 12–14]; however, the factors that cause CCNB1 upregulation remain largely unknown.

Ubiquitin-specific protease 22 (USP22) belongs to a family of more than 70 deubiquitinases in mammals. It contains an N-terminal zinc-finger domain and a ubiquitin-specific peptidase domain at its C terminus [15]. *USP22* has also been identified as a death-from-cancer signature gene [2, 3]. Recent studies demonstrated that USP22 is a *bona fide* component of the deubiquitinating module of the mammalian SAGA (Spt-Ada-Gcn5-Acetyltransferase) complex [16–18] and is involved in transcriptional regulation, cell cycle progression, protein degradation and embryonic stem cell differentiation [19–25]. USP22 is highly expressed in many types of human cancers, including colon cancer [26]; however, the physiological functions of USP22 and its role in tumorigenesis, as well as the underlying molecular mechanisms resulting in both normal and abnormal cell cycle regulation, are largely unknown. We recently discovered a novel molecular mechanism by which USP22 affects apoptosis: USP22 antagonizes p53 transcriptional activation by stabilizing SIRT1 [25]. How USP22 regulates cell cycle progression and how USP22 is regulated remains unclear.

Here, we demonstrate that crosstalk between the two putative cancer stem cell genes, *USP22* and *CCNB1*, has an important role in colorectal tumorigenesis. USP22 regulates CCNB1 protein stability to promote cell cycle progression, as well as cancer cell growth, when it is aberrantly upregulated. Notably, USP22-mediated CCNB1 deubiquitination is regulated by CDK1, a kinase that requires physical association with CCNB1 to achieve full enzymatic activity to control cell cycle progression. USP22 is degraded by the APC/C E3 ubiquitin ligase complex, which also targets CCNB1 for destruction [5, 27], thereby allowing cells to exit from mitosis, presumably by facilitating CCNB1 degradation. Furthermore, we find a positive correlation between USP22 and CCNB1 expression in human colon cancers.

## Results

### USP22 is overexpressed in human colon cancers and positively correlates with CCNB1 protein level

**Figure 1:**
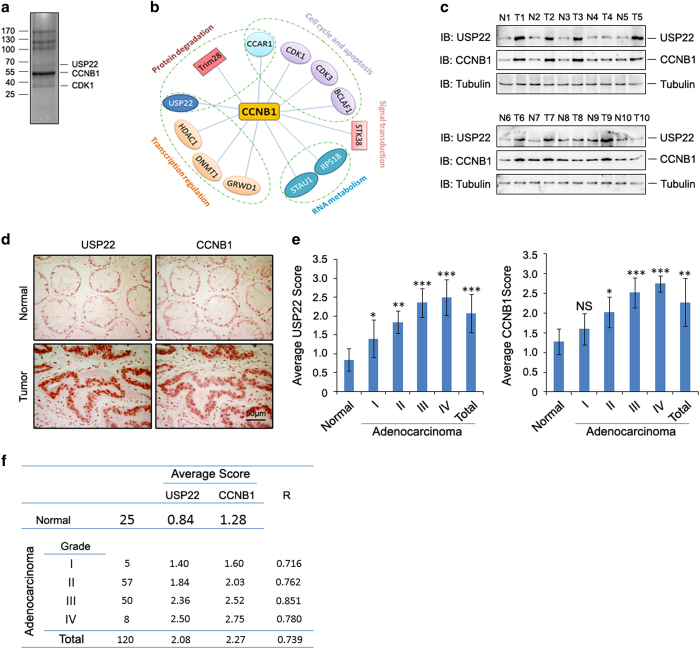
Both ubiquitin-specific protease 22 (USP22) and cyclin B1 (CCNB1) are upregulated in human colon cancers. (**a**) Flag-CCNB1 pull-down products from affinity purification using HCT116 cells were separated by SDS–PAGE and visualized by coomassie brilliant blue staining. (**b**) Interaction network of CCNB1-associated proteins. (**c**) The lysates of frozen colon tumor tissues (T) and their adjacent normal colon controls (N) were subjected to immunoblotting analysis with antibodies to USP22, CCNB1 and tubulin. Samples from 10 patients were analyzed. (**d**) The expression levels of USP22 and CCNB1 in normal human colon tissues (top) and colon cancer tissues (bottom) were determined by immunohistochemistry (IHC) staining with specific antibodies. Representative images are shown. (**e**) The expression levels of USP22 and CCNB1 in micro tissue array (MTA) slides of paraffin-fixed normal human colon or colon tumor tissue were determined by IHC with specific antibodies, and then scored and analyzed; *P<0.05; **P<0.01; ***P<0.001. NS, no significant difference. (**f**) The correlation of USP22 and CCNB1 protein expression was analyzed by IHC staining in normal and human colon cancer tissues.

Elevated levels of CCNB1, in particular its nuclear accumulation, indicate more aggressive cancer and poor prognosis [8, 28]; however, the contributing factors remain to be identified. To study how the functions of CCNB1 are regulated, we used a proteomic approach and identified CCNB1-interacting proteins (Figure 1a and Supplementary Figure S1a). A total of 12 proteins were identified (Figure 1b). To confirm the interactions between CCNB1 and its putative partners, we selectively tested the interactions between CCNB1 and seven interaction candidates. Except for GRWD1 (Supplementary Figure S1h), the interactions between CCNB1 and the other six candidates were confirmed in transiently transfected HCT116 cells (Supplementary Figure S1b–g), confirming that these proteins are true CCNB1 interactors. Together with the fact that CDK1 and CDK3 were previously reported as CCNB1 interactors [29], these results indicate that our proteomic approach yielded highly specific and reliable identification of CCNB1-binding proteins. CCNB1 appears to interact with proteins involved in a wide spectrum of cellular functions, including protein degradation, transcriptional regulation, RNA processing, signal transduction and cell cycle regulation (Figure 1b).

Interestingly, USP22, a ubiquitin-specific peptidase that protects its interaction partners from ubiquitination-mediated degradation [23, 25], was identified as one of the CCNB1 interactors (Figure 1a and b and Supplementary Figure S1b). Next, we questioned whether USP22 is involved in the elevated CCNB1 protein expression in human colon cancers. In fact, immunoblotting analysis confirmed dramatically higher protein expression levels of both CCNB1 and USP22 in colon cancer tissues (in 7 of 10 patients) compared with those in adjacent normal controls (Figure 1c). Immunohistochemistry staining detected that both USP22 and CCNB1 proteins were dramatically increased in more than 65% of human colon cancers and a strong positive correlation between CCNB1 and USP22 protein expression (average *R*=0.739) was found in human colon cancer tissues compared with those in normal human colons (Figure 1d–f). Therefore, these results suggest that crosstalk between USP22 and CCNB1 is associated with human colon cancer development.

### USP22 interacts with CCNB1

**Figure 2:**
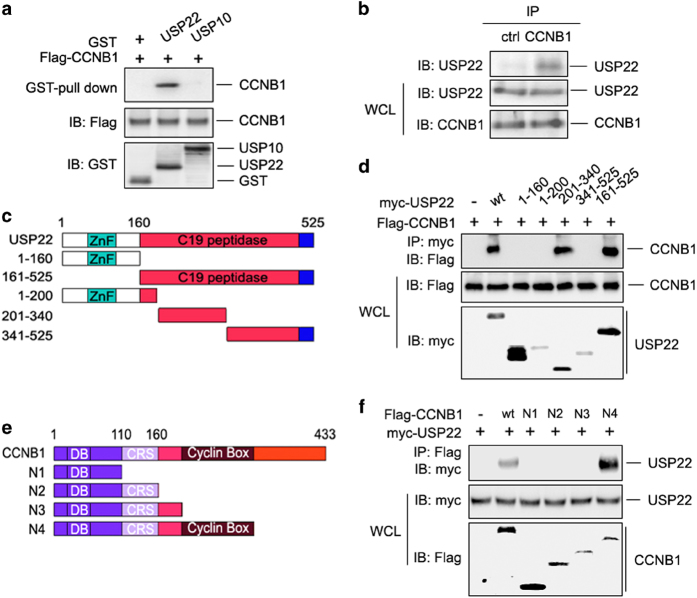
USP22 interacts with CCNB1. (**a**) HCT116 cells were transfected with Flag-tagged CCNB1 expression plasmid. After 48 h, cells were lysed and incubated with GST-USP22 or GST-USP10 and GSH-Sepharose as well. Proteins retained on Sepharose were then blotted with the indicated antibodies. (**b**) HCT116 cells were lysed, and the interaction between endogenous CCNB1 and USP22 was determined by immunoprecipitation of CCNB1 using normal rabbit IgG as control and immunoblotting with anti-USP22 antibody (top panel). (**c**) Domain structures of USP22 and its truncated mutants. USP22 contains an N-terminal zinc-finger domain and a C-terminal C19 peptidase catalytic domain. (**d**) CCNB1 expression plasmids were co-transfected with USP22 or each of the truncated mutants shown in **c** into HCT116 cells. The interaction between USP22 or its mutants and CCNB1 was examined. (**e**) Domain structures of CCNB1 and its truncated mutants. CCNB1 contains an N-terminal destruction box (DB), followed by a cytoplasmic retention sequence (CRS) and a cyclin box domain. (**f**) Myc-USP22 expression plasmids were co-transfected with CCNB1 or each of the truncated mutants shown in **e** into HCT116 cells. The interaction of CCNB1 or its mutants was examined. CCNB1, cyclin B1; IgG, immunoglobulin G; USP2, ubiquitin-specific protease 22.

To study the underlying molecular mechanisms of USP22/CCNB1 crosstalk in colon cancer development, we first confirmed their interaction (Supplementary Figure S1b). The interaction is specific, as CCNB1 was pulled down by the GST-USP22 fusion protein, but not GST-USP10 fusion protein (Figure 2a). The interaction between endogenous USP22 and CCNB1 in human colon cancer cells was also detected, as anti-CCNB1-specific antibody but not normal mouse immunoglobulin G immunoprecipitated USP22 protein (Figure 2b). Thus, our study identified USP22 as an interacting protein of CCNB1 in human colon cancer cells. To further refine our understanding of the molecular interaction between USP22 and CCNB1, we generated truncated USP22 mutants (Figure 2c). Co-immunoprecipitation (co-IP) and immunoblotting experiments showed that the N-terminal portion of the USP22 C19 peptidase domain mediated the interaction with CCNB1 (Figure 2d). Similarly, truncated CCNB1 mutants were generated (Figure 2e), and we discovered that the cyclin box of CCNB1 mediated its interaction with USP22 (Figure 2f). Therefore, USP22 is an interacting partner of CCNB1 in human colon cancer cells.

### USP22 stabilizes CCNB1 by negatively regulating its ubiquitination

**Figure 3:**
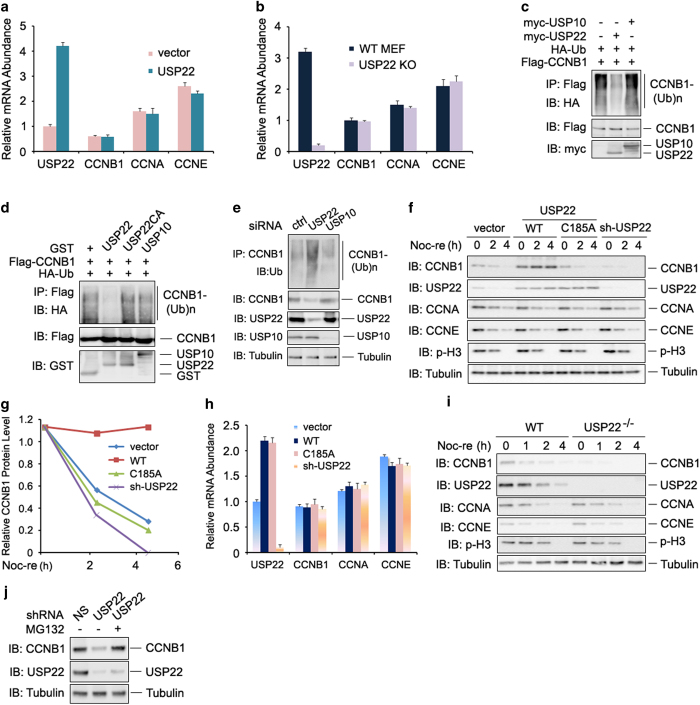
USP22 deubiquitinates and stabilizes CCNB1. (**a** and **b**) The mRNA levels of USP22, CCNB1, CCNA and CCNE in transfected HCT116 cells (**a**) or MEF cells (**b**) were analyzed by real-time PCR. Error bars represent data from three independent experiments. (**c**) Flag-CCNB1 and HA-Ub expression plasmids were co-transfected with empty vector, myc-USP22 or myc-USP10 into HCT116 cells. CCNB1 ubiquitination was determined by immunoprecipitation of CCNB1 with anti-Flag antibodies and immunoblotting with anti-HA antibody. (**d**) Deubiquitination of CCNB1 by USP22 *in vitro*. Ubiquitinated CCNB1 was purified from HCT116 cells transiently transfected with Flag-CCNB1 and HA-Ub expression plasmids, and the cell lysates were incubated with indicated GST or GST-fusion proteins at 37 °C for 2 h and then subjected to SDS–PAGE analysis. CCNB1 ubiquitination levels were determined by immunoblotting with anti-HA antibody. (**e**) Knockdown of USP22 by siRNA in HCT116 cells promotes CCNB1 ubiquitination. HCT116 cells were transfected with indicated siRNA and ubiquitination assay was analyzed as in **c**. (**f**) HCT116 cells stably expressing USP22, its C185A mutant, or USP22 shRNA were synchronized by treatment of nocodazole for 12 h, then mitotic cells were collected by shake-off approach and released into fresh medium for the indicated times. Levels of indicated proteins were detected by corresponding antibodies. Tubulin was used as loading control. (**g**) The band densities of CCNB1 in **f** were analyzed using a Bio-Rad imaging software. (**h**) The mRNA levels of USP22, CCNB1, CCNA and CCNE in the established HCT116 stable cell lines were analyzed as in **a**). (**i**) Loss of USP22 facilitates CCNB1 degradation in MEF cells. The indicated proteins in wild-type or USP22 knockout MEF cells were analyzed as in **f**. (**j**) HCT116 cells stably expressing control or USP22-specific shRNA were treated with or without MG132 (20 μm) for 2 h before harvested. The expression levels of CCNB1, USP22 and tubulin were analyzed by immunoblotting. CCNA, cyclin A; CCNB1, cyclin B1; CCNE, cyclin E; MEF, mouse embryonic fibroblast; shRNA, short hairpin RNA; siRNA, small interfering RNA; USP2, ubiquitin-specific protease 22.

Previous studies suggested that USP22 is involved in transcriptional regulation [21] and protein degradation [23, 25]. Neither gain nor loss of USP22 function affected the mRNA levels of CCNB1, cyclin A (CCNA) or cyclin E (CCNE; Figure 3a and b), excluding the possibility that USP22 regulates CCNB1 functions at the transcriptional level. We then hypothesized that USP22 may regulate the cell cycle through deubiquitinating and stabilizing CCNB1. Indeed, USP22 inhibited CCNB1 ubiquitination both *in vivo* and *in vitro* (Figure 3c and d and Supplementary Figure S2a). Conversely, USP22 knockdown resulted in elevated CCNB1 ubiquitination (Figure 3e). As a negative control, neither overexpression nor knockdown of USP10 had any effect on CCNB1 ubiquitination (Figure 3c–e). Therefore, USP22 is a CCNB1-specific deubiquitinase. The deubiquitinase catalytic activity of USP22 is required for CCNB1 deubiquitination because the catalytically inactive USP22 (USP22/C185A) mutant failed to suppress CCNB1 ubiquitination without affecting its interaction with CCNB1 (Supplementary Figure S2b and S2c).

Ubiquitination of CCNB1 protein has been found to promote its degradation [30–32]. To test whether USP22 regulates CCNB1 protein stability, we established stable cell lines with either overexpression or knockdown of USP22. We tested the knockdown efficiency of different short hairpin RNAs (shRNAs) against USP22 and selected shRNA #3 to generate a USP22 knockdown stable cell line (Supplementary Figure S3). CCNB1 is a master regulator of G2/M transition and its levels peak during mitosis. Therefore, we isolated USP22-knockdown HCT116 cells arrested in mitosis by mitotic shake-off after nocodazole treatment. After releasing into fresh media, the levels of both CCNB1 and phosphorylated histone H3 were gradually reduced during the synchronized exit of cells from M phase, as expected [33]. Notably, stable expression of USP22, but not its C185A mutant, protected CCNB1 from degradation (Figure 3f and g) without affecting its mRNA expression levels (Figure 3h) during cell exit from M phase. We further demonstrated that loss of *usp22* expression resulted in the instability of CCNB1 (Figure 3i), while the mRNA expression level of *ccnb1* was not affected by *usp22* deficiency (Figure 3b). Moreover, the proteasome inhibitor MG132 protected CCNB1 from degradation in *usp22 *knockdown cells (Figure 3j), implying that USP22 mediates CCNB1 stabilization through regulating the proteasomal pathway. As CCNB1 regulates cell cycle progression by interacting with and activating CDK1[5], we asked whether USP22, which has been shown to promote cell cycle progression [22], regulates CDK1 activation through CCNB1 stabilization. As shown in Supplementary Figure S4c, the level of phosphorylated histone H1, a well-known substrate of CCNB1/CDK1[ 34], is increased upon USP22 overexpression. Collectively, these results indicate that USP22-mediated deubiquitination protects CCNB1 from degradation.

### CDK1 phosphorylates USP22 to optimize its activity in CCNB1 stabilization during the G2/M phase

**Figure 4:**
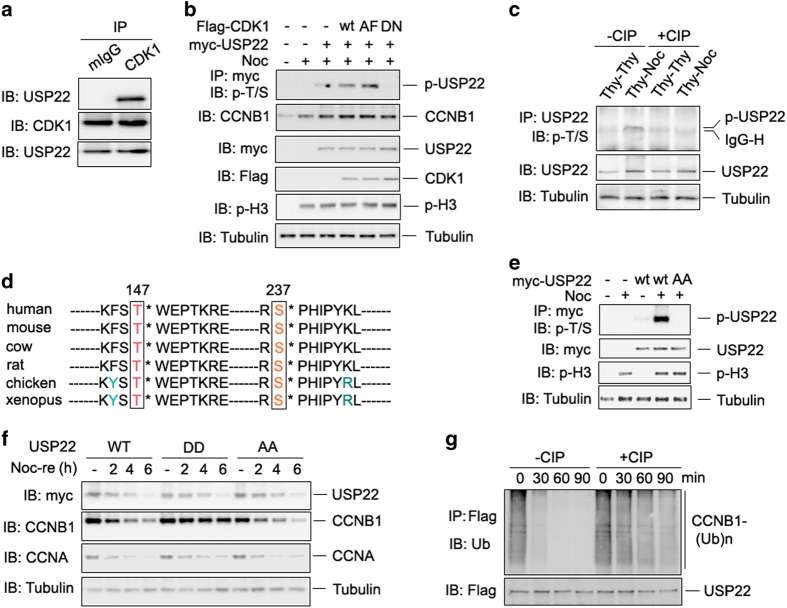
Cyclin-dependent kinase 1 (CDK1) phosphorylates ubiquitin-specific protease 22 (USP22) to promote its deubiquitinase activity. (**a**) The endogenous interaction between CDK1 and USP22 was analyzed as in Figure 2b. (**b**) Myc-USP22 plasmids were co-expressed with wild-type (WT), the constitutively active form (AF) of CDK1, or the kinase-inactive D146N mutant of CDK1. After 24 h, cells were treated without or with nocodazole for 12 h. USP22 phosphorylation in the lysates of transfected cells was analyzed. (**c**) HCT116 cells were treated with double thymidine (Thy–Thy) or with thymidine followed by nocodazole (Thy–Noc). The cell lysates were treated with or without calf intestinal alkaline phosphatase (CIP) for 1 h as indicated. USP22 phosphorylation in the lysates of treated cells was analyzed as described in **b**. (**d**) Conserved T147 and S237 amino acids of USP22 in each indicated species are shown. (**e**) WT USP22 or its phosphorylation-defective mutant USP22/AA was co-expressed in HCT116 cells. Their phosphorylation was determined as in **b**. (**f**) HCT116 cells stably expressing WT USP22 or its phosphomimetic mutant (USP22/DD) or phosphorylation-defective mutant USP22/AA were synchronized in prometaphase with Thy and Noc treatment, then released into fresh medium for the indicated times (Noc-re). (**g**) HA-ubiquitin-conjugated Flag-CCNB1 was affinity purified from co-transfected HCT116 cells. Flag-USP22 was purified from HCT116 cells and treated with or without CIP for 1 h. Ubiquitinated CCNB1 was then mixed with USP22 for indicated time and analyzed by immunoblotting.

CDK1, formerly called Cdc2 [35, 36], interacts with CCNB1 to form an active heterodimer and to function as a mitosis-promoting factor whose activity determines the timing of mitosis [4]. From our proteomic analysis, we noticed that CCNB1 appears to form a complex with both USP22 and CDK1; this prompted us to ask whether CDK1 phosphorylates USP22. At first, we confirmed the interaction of USP22 with CDK1 by co-IP and immunoblotting (Figure 4a). We then analyzed whether USP22 is phosphorylated by CDK1. Only weak USP22 phosphorylation was detected in HCT116 cells, presumably catalyzed by endogenous CDK1, and CDK1 overexpression significantly increased USP22 phosphorylation. In addition, expression of the constitutively active form of CDK1 (CDK1/AF) [37, 38] further enhanced USP22 phosphorylation in HCT116 cells (Figure 4b). Moreover, mutation of the aspartic acid at position 146 of CDK1 (CDK1/D146N), which is a kinase-inactive mutant [39], completely abolished its ability to promote USP22 phosphorylation (Figure 4b). In an *in vitro* kinase assay, co-incubation of the purified GST-USP22 fusion proteins and the constitutive forms of CDK1 (CDK1/AF) immunoprecipitated from transiently transfected HCT116 cells confirmed USP22 phosphorylation by CDK1 (Supplementary Figure S4d). Endogenous USP22 was phosphorylated in G2/M phase, but not in G1 phase, as the phosphorylated forms of USP22 were detected in cells treated with thymidine-nocodazole but not double thymidine (Figure 4c). These findings show that CDK1 is a kinase that catalyzes USP22 phosphorylation in a cell cycle-specific manner.

Proteomic analysis of the USP22 proteins from cells expressing the constitutively active forms of CDK1 identified two phosphorylated amino acids of USP22, S237 and T147 (Supplementary Figure S4a and S4b), both of which are conserved amino acids from Xenopus to human (Figure 4d). These results indicate that T147 and S237 are the potential CDK1 phosphorylation sites and imply that these residues have important functions in CDK1-mediated USP22 phosphorylation. Replacing both T147 and S237 with alanine (AA) completely abolished USP22 phosphorylation and led to decreased CCNB1 protein levels even when CDK1 was co-expressed in HCT116 cells (Figure 4e and Supplementary Figure S4e). In addition, *in vitro* kinase assays confirmed that T147 and S237 are the phosphorylation sites of USP22 because co-incubation with the constitutively active form of CDK1 failed to induce USP22/AA phosphorylation (Supplementary Figure S4d).

CCNB1 protein expression levels peak in G2/M phase and are degraded by the APC E3 ubiquitin ligase complex to allow the exit from M phase [5, 27, 40, 41]. To study the cell cycle-specific functions of CDK1-mediated USP22 phosphorylation, in addition to the phosphorylation-defective USP22/AA mutant, we generated a phosphorylation-mimetic mutant by replacing both T147 and S237 with aspartic acid (D) (USP22/DD). HCT116 cells stably expressing either wild-type or its DD or AA mutant were synchronized with thymidine and nocodazole at prometaphase (Figure 4f) or cultured in normal media (Supplementary Figure S4f). As expected, CCNB1 protein was unstable in HCT116-USP22/AA cells. In contrast, the degradation of CCNB1 was blocked in HCT116-USP22/DD cells, suggesting that CDK1-mediated USP22 phosphorylation enhances its ability to stabilize CCNB1 (Figure 4f) and may be involved in mitotic progression (Supplementary Figure S4f). To test our hypothesis, we purified USP22 protein from HCT116 cells, treated the protein with calf intestinal alkaline phosphatase, which presumably suppresses USP22 phosphorylation, and then co-incubated it with ubiquitinated CCNB1 protein. Treatment significantly impaired the ability of USP22 to suppress CCNB1 ubiquitination compared with untreated control (Figure 4g). Therefore, USP22 phosphorylation is correlated with its cell cycle-specific deubiquitinase activity.

### Mitotic degradation of USP22 is mediated by its putative destruction box and APC^CDC20^

**Figure 5:**
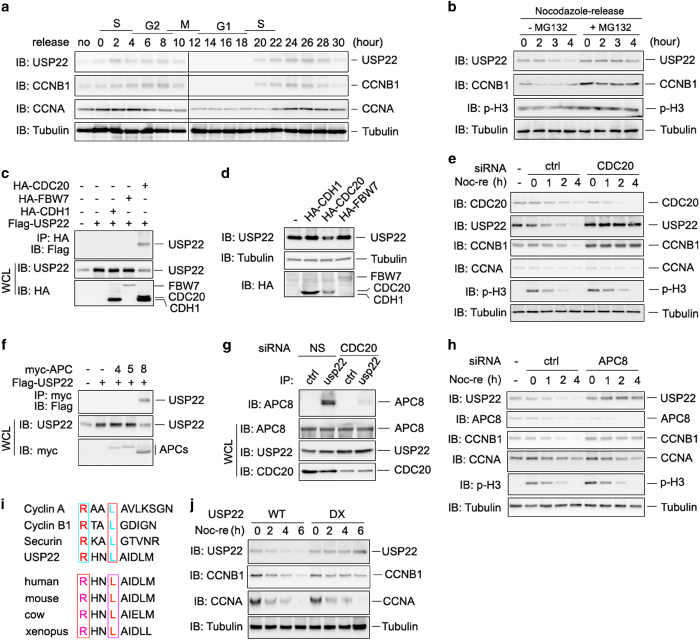
APC^CDC20^ destructs USP22 protein during cell exit from M phase. (**a**) HCT116 cells were arrested at the G1/S boundary with a double thymidine treatment and then released into fresh medium. Cells were collected every 2 h and lysates were analyzed by immunoblotting with indicated antibodies. (**b**) HCT116 cells were synchronized in prometaphase with thymidine and nocodazole, then released into fresh medium without or with 20 μm MG132 treatment for the indicated durations. (**c**) USP22 specifically interacts with CDC20 but not with FBW7 or CDH1. HCT116 cells were transfected with indicated plasmids and USP22 interactions with each of them were analyzed as in Supplementary Figure S1B. (**d**) CDH1, CDC20 or FBW7 expression plasmids were transfected into HCT116 cells. The endogenous levels of USP22 protein in transfected cells were analyzed by immunoblotting (top panel). (**e**) CDC20 is required for USP22 destruction in mitosis. *Cdc20* was depleted from HCT116 cells using siRNA, cells were synchronized by treatment of nocodazole for 12 h, then mitotic cells were collected by the shake-off approach and released into fresh medium for the indicated times. Levels of indicated proteins were detected by corresponding antibodies. Tubulin was used as a loading control. (**f**) USP22 interacts with APC8. USP22 plasmids were co-transfected without or with each of the indicated APC proteins. Their interactions were determined as described in **c**. (**g**) HCT116 cells were transfected with control or CDC20-specific siRNA. The interaction of USP22 with APC8 was examined as in **c**. (**h**) APC8 is required for USP22 destruction in mitosis. APC8 was depleted from HCT116 cells using siRNA, then cells were synchronized and analyzed as in **e**. (**i**) Sequence alignment of putative USP22 D-box motif compared with those from cyclin A, cyclin B1 and securin. The two residues in USP22 mutated to alanine to yield the USP22 D-box mutant (R98A/L101A) are indicated (upper panel). The conserved D-box sequence of USP22 is indicated (lower panel). (**j**) The USP22 D-box mutant is stable in cells arrested in mitosis. HCT116 cells stably expressing either wild-type USP22 (WT) or the D-box mutant were synchronized and analyzed as in **e**. APC, anaphase-promoting complex; siRNA, small interfering RNA; USP2, ubiquitin-specific protease 22.

We noticed that USP22 protein is also gradually degraded after cells were released to exit mitosis by changing to nocodazole-free medium (Figure 4f). To characterize the expression profile of USP22 protein during cell cycle progression, HCT116 cells were arrested at the G1/S boundary by double thymidine treatment and then released into fresh media. The cell cycle was analyzed (Supplementary Figure S5) and the endogenous expression of both USP22 and CCNB1 proteins fluctuated in a similar pattern, with their expression levels peaking at G2/M phase followed by a gradual decline when cells exited from the M phase (Figure 5a). As a control, CCNA protein levels peaked at S phase and declined as cells entered into G2/M phase (Figure 5a). These results imply that USP22 expression is regulated during cell cycle progression and its regulation is associated with CCNB1 protein expression levels (Figure 5a). Similar to CCNB1, USP22 protein expression is likely regulated by ubiquitin-proteasome pathway because USP22 remained stable in nocodazole-synchronized HCT116 cells after MG132 treatment (Figure 5b). Given that degradation of CCNB1 is required for cells to exit M phase and enter anaphase [42, 43], we proposed that USP22 degradation, which presumably allows CCNB1 degradation during late M phase, is necessary for cell cycle regulation.

To identify the E3 ligase that degrades USP22, we tested the interaction between USP22 and FBW7, CDC20, and CDH1, all of which are active during exit from mitosis [44, 45]. We detected a strong interaction between USP22 and APC/C adapter protein CDC20 (Figure 5c and Supplementary Figure S6a), which is required to initiate chromatid separation and entrance into anaphase [46, 47]. This interaction was specific because USP22 interaction with either CDH1 or FBW7 was not detected (Figure 5c). Further analysis using the truncated CDC20 mutants identified that the WD40 repeat-containing C terminus of CDC20 mediated its interaction with USP22 (Supplementary Figure S6b and S6c). In addition, we discovered that the N-terminal region of USP22 composed of 160 amino acids was sufficient to mediate its interaction with CDC20 (Supplementary Figure S6d). These results suggest that APC/CDC20 E3 ligase is possibly involved in USP22 destruction during cell cycle progression.

We noticed that the expression of CDC20, but not CDH1 or FBW7, significantly inhibited USP22 protein expression (Figure 5d), implying the CDC20-containing APC/C E3 ligase complex is involved in regulating USP22 protein stability in HCT116 cells, in particular during the transition period from M to G0 phase during cell cycle progression. To confirm this, HCT116 cells arrested at G2/M phase were collected by shake-off during nocodazole treatment and then released into fresh culture media at different time points (Figure 5e and h). Immunoblotting analysis of the levels of phosphorylated histone H3 confirmed the transition of HCT116 cells from M to G0/G1 phase. During the M to G0/G1 transition, both USP22 and CCNB1 proteins were gradually degraded, indicating that, like CCNB1, USP22 is degraded during the M to G0/G1 transition. Notably, knockdown of endogenous CDC20 led to USP22 stabilization (Figure 5e), indicating that CDC20 regulates USP22 protein stability during cell cycle progression. In addition, APC8, one subunit of APC/C and a protein required for CCNB1 destruction in metaphase [27], was identified to interact with USP22 (Figure 5f). This interaction likely occurs through CDC20, since knockdown of CDC20 inhibited USP22 interaction with APC8 (Figure 5g). To further support our hypothesis, knockdown of APC8 expression, presumably disrupting APC/C E3 ligase activity, dramatically increased the stability of endogenous USP22 protein in HCT116 cells (Figure 5h). Therefore, APC/CDC20 E3 ligase complex promotes USP22 protein degradation, presumably allowing CCNB1 degradation for cells to exit M phase.

The APC/C E3 ubiquitin ligase targets degradation of cyclins and other substrates by recognizing their destruction box (D-box) motifs [48, 49]. As a potential substrate of CDC20-containing APC/C E3 ligase, USP22 may contain a D-box for its degradation during cell cycle progression. Indeed, a well-conserved putative D-box motif does exist in USP22 protein (Figure 5i) and this D-box is located in the N-terminal region of USP22 that we identified to interact with CDC20 (Supplementary Figure S6d). We then asked whether the D-box mediates USP22 degradation by APC/C E3 ubiquitin ligase during mitotic exit. As indicated in Figure 5j, mutation of the conserved amino acids in the D-box of USP22 (USP22/DX) prevented mutant USP22 protein degradation during nocodazole-induced mitotic arrest. As a deubiquitinase, USP22 may regulate the protein stability of all interacting partners. However, USP22 expression did not affect the protein expression levels of CDK1, CDC20 and APC8, or changes in their mRNA expression levels in USP22-null mouse embryonic fibroblasts (Supplementary Figure S7), excluding the possibility that USP22 regulates CDK1, CDC20 and APC8 expression. Therefore, our discovery indicates that the APC/CDC20 E3 ligase regulates USP22 protein stability in a cell cycle-dependent manner.

### USP22 downregulation mitigates colon cancer development

**Figure 6:**
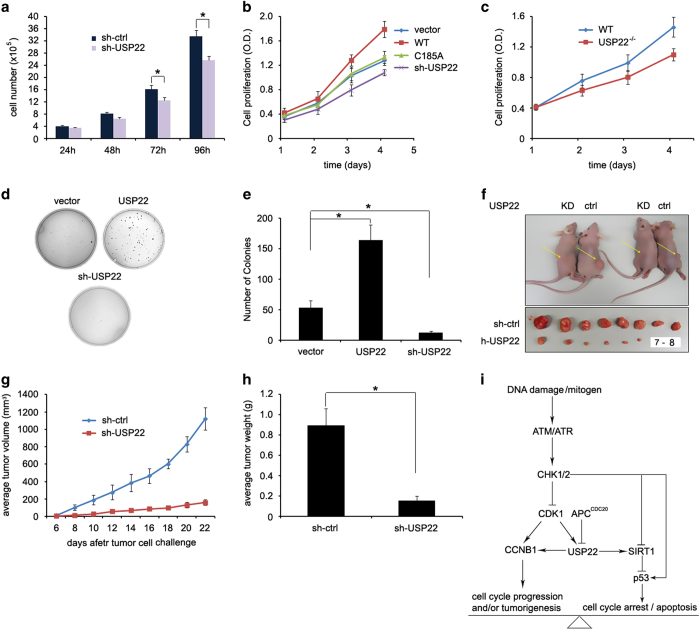
Knockdown of USP22 by shRNA inhibits colorectal tumorigenesis. (**a**) 2×10^5^ cells were seeded in six-well plates. The growth of cells was examined by counting cell numbers every 24 h after plating. Error bars represent data from three independent experiments; *P*<0.05. (**b**) The growth of stably expressing HCT116 cells indicated plasmids was examined by WST-1 assay as described in materials and methods. (**c**) Wild-type or *usp22*-null MEF cells were subjected to WST-1 assay as in **b**. (**d**) Stably expressing HCT116 cells indicated plasmids were seeded in soft agar and cultivated for 3–4 weeks. Colony formation was assayed by light microscopy and representative images are shown. (**e**) The number of colonies in each plate was counted. Error bars represent data from three independent experiments with a total of three plates per group. *P*<0.05. (**f**–**h**) 1×10^6^ HCT116 or stable USP22 knockdown (KD) cells were injected subcutaneously into nude mice (*n*=8 per group). Twenty-two days after injection, two pairs of representative mice were photographed (**f**, top panel). Tumors were isolated and photographed. 7–8 represents no tumors (**f**, bottom panel). The tumor sizes were measured every other day, and their growth curves are shown (**g**). Mice were euthanized at day 22, and the tumors were excised and weighed at the end of the experiment (**h**). *P*<0.05. (**i**) Our working model of CCNB1 regulation by USP22. CCNB1, cyclin B1; shRNA, short hairpin RNA; siRNA, small interfering RNA; USP2, ubiquitin-specific protease 22.

We have demonstrated that USP22 regulates the cell cycle by stabilizing CCNB1. To further support our observation that USP22 promotes cell cycle progression and probe further into its potential role in cancer development, we first examined its function in cell proliferation. We detected a statistically significant reduction in cell proliferation of HCT116 cells with stable knockdown of USP22 (Figure 6a and b) or *usp22*^−^^/−^ mouse embryonic fibroblast cells (Figure 6c) compared with wild-type controls. Thus, USP22 is involved in cell proliferation possibly through promoting CCNB1 stabilization.

Elevated levels of both CCNB1 and USP22 indicate more aggressive cancer and a poor prognosis [8, 28] and both of these proteins were upregulated in colon cancer patients (Figure 1c–f). Hence, we determined whether downregulation of USP22 suppresses colon cancer cell growth and cancer progression. As indicated in Figure 6d and e, a soft agar assay revealed that stable expression of USP22 dramatically enhanced the colony formation of HCT116 cells while USP22 knockdown resulted in fewer colonies, suggesting that USP22 plays an important role in colon cancer cell growth. Next, we used a xenograft colon cancer model, as described previously [50–52]. USP22 knockdown significantly inhibited tumor growth in nude mice (Figure 6f). Both the growth speed and average tumor size were dramatically reduced in mice that had been injected with USP22-knockdown HCT116 cells (Figure 6g and h). Therefore, USP22 expression is critical for colon cancer growth, as tested both *in vitro* and in the xenograft mouse model. Our data indicate a great therapeutic potential of USP22 suppression in human colon cancer therapy.

Based on our discoveries here and in a previous report [25], we propose a model for the tumorigenic activity of USP22 (Figure 6i). On the one hand, USP22 is a CCNB1 deubiquitinase that promotes cell cycle progression and colon cancer cell growth. USP22-mediated CCNB1 stabilization is regulated by both CDK1 and the APC/C E3 ubiquitin ligase complex. CDK1 phosphorylates USP22 to enhance its activity in CCNB1 stabilization during G2/M phase. In contrast, the APC/CDC20 E3 ligase complex negatively regulates USP22 activity by targeting it for degradation, presumably allowing CCNB1 downregulation so that cells can exit mitosis and enter anaphase. On the other hand, USP22 suppresses the pro-apoptotic and cell cycle arrest activity of p53 through SIRT1 [25]. As a consequence, gain of USP22 functions promotes cell cycle progression and inhibits cell apoptosis, leading to cancer cell hyper-proliferation and tumorigenesis.

## Discussion

Our study demonstrates that USP22 is a CCNB1 deubiquitinase and overexpression of both proteins may be correlated with colorectal tumorigenesis. This conclusion is supported by the following observations. First, both USP22 and CCNB1 protein expression are elevated with a strong positive correlation in human colon cancer tissues. Second, USP22 is a CCNB1 interactor and a deubiquitinase, which negatively regulates CCNB1 ubiquitination and stabilizes CCNB1 during the G2/M phase to promote cell cycle progression. Third, USP22 is phosphorylated by CDK1 during the G2/M phase of the cell cycle and this phosphorylation optimizes the enzyme activity of USP22 to deubiquitinate CCNB1. Fourth, USP22 is ubiquitinated and degraded by CDC20-containing APC/C complex during cell exit from M phase, presumably to release the brake on CCNB1 degradation. This is important because CCNB1 degradation is necessary for cells to exit mitosis and to enter anaphase during cell cycle progression. When USP22 is abnormally upregulated (due to APC/C inactivation or unknown reason), CCNB1 level is elevated and it possibly causes aberrant cycle progression and disease progression. Finally, USP22 promotes tumor formation *in vitro* and in xenograft nude mice.

Recently, many genes have been identified as prognostic signatures in colon cancer [3, 53–56]. Previous and recent data suggested that both USP22 and CCNB1 are members of these signature gene families, the upregulation of which is associated with the development, progression and metastasis as well as chemotherapeutic resistance of several types of human cancers [57]. Upregulation of either CCNB1 or USP22 has been found in human colon cancers [8, 9]. Our discovery here, that both USP22 and CCNB1 proteins are elevated and positively associated in human colon cancers, indicates that USP22-mediated CCNB1 stabilization is one possible molecular mechanism underlying its proto-oncogenicity. Most importantly, our observation that USP22 knockdown impairs human cancer cell growth and tumor formation suggests that USP22 is involved in tumorigenesis, implying a great therapeutic potential of USP22 suppression in colon cancer treatment. As both USP22 and CCNB1 are upregulated and positively correlated in colon cancer, targeting either of them might be a good approach to cancer therapy. However, CCNB1 is a regulatory subunit of CDK1 and is one of the main cyclin family members, it is not convenient to be directly manipulated in anti-cancer therapy. Thus, targeting USP22 might be an effective and convenient approach to treat cancers with high levels of CCNB1 expression. Notably, small molecule inhibitors that specifically antagonize several other USPs have been identified, some of which show potent activity in suppressing tumor cell growth [58–60]. Currently, we are developing small molecule inhibitors to target USP22 in cancer treatment.

CCNB1 expression is tightly regulated during cell cycle progression. Its expression levels peak during G2/M phase but are quickly degraded during late M phase to allow cells to exit from mitosis [4, 5, 27, 40, 41]. Two ubiquitin ligase complexes, the SKP1-CUL1-F-box-protein (SCF) and APC/C, are responsible for the degradation of cell cycle regulators. APC/C is a CRL (cullin RING ubiquitin ligase)-like ligase, which uses CDC20 and CDH1 as co-activators in mitotic cell cycle regulation [27, 61]. In particular, the Cdk1-APC/C cell cycle oscillator circuit functions as a time-delayed, ultrasensitive switch during cell cycle progression[62]. Gene alterations in several components of the APC/C complex, including APC6/CDC16 and APC8/CDC23, have been found in human colon cancers [9]. The abnormal levels of APC/C targets such as CCNB1 lead to dysregulation of the cell cycle progression of colon epithelial cells through mitosis [9]. Our study demonstrates that USP22 is also a substrate of the APC/C E3 ligase complex, which degrades USP22 during cell exit from M phase. Similar to the destruction of other substrates, the APC/C recognizes the D-box of USP22 through its adapter protein CDC20 for USP22 degradation. To our best knowledge, the APC/C is the first E3 ligase of USP22 that controls the protein expression levels of USP22 in a cell cycle-dependent manner. In addition, given the fact that USP22 is abnormally upregulated and there are gene alterations in several components of the APC/C complex in human colon cancers [9], it will be interesting to investigate whether these two events are positively associated. Based on our discovery, it will not be surprising to add USP22 in the list of the abnormally upregulated APC/C targets in human cancers.

USP22-mediated CCNB1 deubiquitination appears to be necessary to promote cell cycle progression. Interestingly, USP22 activity is regulated by CDK1, which catalyzes USP22 phosphorylation to elevate USP22 ability in CCNB1 deubiquitination and stabilization. Therefore, on one hand, CDK1 directly regulates cell cycle progression by associating with CCNB1 to achieve full activity. On the other hand, CDK1 enhances USP22 activity to stabilize CCNB1 during the G2/M phase. However, the detailed molecular mechanism by which CDK1-mediated phosphorylation enhances USP22 deubiquitinase activity remains to be investigated. Since one of the phosphorylation sites, S237, is located in the C19 peptidase domain, CDK1-mediated phosphorylation might cause conformational changes to optimize its catalytic activity. In addition, as S237 is also within the portion of USP22 that interacts with CCNB1, it is possible that CDK1-mediated S237 phosphorylation enhances USP22 interaction with CCNB1. Moreover, S237 phosphorylation might recruit unknown co-factors that facilitate USP22-mediated CCNB1 deubiquitination.

In addition to CCNB1, we have recently shown that USP22 antagonizes p53 functions by stabilizing the NAD-dependent deacetylase SIRT1 [25], which inhibits p300-induced p53 acetylation and suppresses p53 functions [63]. Our studies suggest that SIRT1 and CCNB1 interact with USP22 through different regions. SIRT1 recognizes the N-terminal zinc-finger domain of USP22, whereas CCNB1 directly binds to the USP22 peptidase catalytic domain. In addition to its role in protecting substrates from protein degradation, USP22 has also been found to regulate transcription by deubiquitinating H2A and H2B as part of the mammalian SAGA complex [21, 22]. Moreover, it has been shown that USP22 promotes cell growth by regulating the far upstream element (FUSE)-binding protein 1 (FBP1), a transcriptional regulator of p21 [64]. As USP22 is a component of the SAGA complex [16, 17], it is very likely that USP22 cooperates with other SAGA components to regulate protein stability and gene transcription during cell cycle progression. Thus, USP22 is possibly involved in promoting cancer cell growth through multiple mechanisms.

In addition to USP22, USP2 has been identified as a specific deubiquitinase that inhibits cyclin D1 ubiquitination by *in vitro* screening of more than 70 deubiquitinases [65], presumably to regulate the G1-to-S phase transition. Therefore, different deubiquitinases appear to regulate the cell cycle in a phase-specific manner by protecting specific cyclin family members from degradation.

## Materials and Methods

### Cells, plasmids and antibodies

Human colon cancer HCT116 cells were cultured in Dulbecco's Modified Eagle's Medium containing 10% fetal bovine serum. *usp22*^+/+^ and *usp22*^−/−^ mouse embryonic fibroblasts were isolated and used as described [20]. USP22 and its mutant plasmids were used as described [20] or cloned into pcDNA3.1. Plasmid DNA that expresses CDC20, CDH1, FBW7 and USP10 were purchased from Addgene company. Plasmids of CCNB1, APC4, APC5 and APC8 are made by PCR and then cloned into PCMV vector. Antibodies and their sources used in this study included anti-USP22 (Sigma Aldrich, St Louis, MO, USA) or as described [16], anti-CCNB1 (for immunohistochemistry), anti-CCNA, anti-CCNE, CDC20, APC8 (Cell Signaling Technology, Danvers, MA, USA), anti-Flag (Sigma Aldrich), anti-Myc, anti-HA, and anti-CCNB1 (for western blotting; Santa Cruz, Inc., Santa Cruz, CA, USA). Anti-phospho-Thr/Ser antibody was purchased from Abcam. Small interfering RNA/shRNA that specifically knocks down USP22 and control small interfering RNA/shRNA were purchased from Open Biosystem company.

### Isolation of CCNB1 interactors by a proteomic approach

HCT116 cells were transfected with Flag-tagged CCNB1 expression plasmids. The transfected cells were lysed with RIPA lysis buffer (150 mm NaCl; 20 mm Tris, pH 7.5; 5 mm EDTA; 1% NP-40; 0.1% SDS and 0.5% sodium deoxycholate) and pre-cleaned by incubating with agarose beads for three times. CCNB1 proteins were immunoprecipitated with anti-Flag antibody-conjugated agarose and the immune complex was eluted from the agarose with 100 μm Flag peptide (Sigma Aldrich). The eluted proteins were digested with trypsin and characterized by mass spectrometry.

### Co-IP and western blotting

Co-IP and western blotting were performed as described [20]. Transiently transfected HCT116 cells were washed with ice-cold phosphate-buffered saline (PBS), resuspended in RIPA lysis buffer with protease inhibitor and incubated on ice for 15 min. Insoluble fractions were removed by centrifugation (15 000 *g*, 15 min). Supernatants were pre-cleaned with protein G-sepharose at 4 °C for 15 min and then incubated with the indicated antibody (1 μg ml^−1^) for 1 h followed by incubation with protein G-sepharose beads for 2 additional hours. The protein G Sepharose beads were washed four times with lysis buffer, dissolved with 4× loading buffer and boiled for 5 min. Supernatants were subjected to SDS–PAGE and transferred to nitrocellulose membrane. After blocking with 5% (w/v) skim milk in Tris-buffered saline containing 0.1% Tween 20, the membrane was incubated overnight at 4 °C with the indicated primary antibodies followed by horseradish peroxidase-conjugated secondary antibody. Membranes were then washed and visualized with enhanced chemiluminescence. When necessary, membranes were stripped using stripping buffer (Bio-Rad, Hercules, CA, USA) and reprobed with corresponding antibodies.

### *In vivo* and *invitro* deubiquitination assay

For *in vivo* ubiquitination assay [20], cells were lysed with ubiquitination buffer containing 1% SDS and boiled at 95 °C for 10 min. The denatured cell lysates were diluted with SDS-negative RIPA buffer to reduce SDS to 0.2% and then subjected to co-IP followed by western blotting with anti-HA or anti-Ub antibodies. The *in vitro* deubiquitination assay was performed as described [62, 63]. Briefly, HCT116 cells were transiently transfected with Flag-CCNB1 and HA-Ub expression plasmids. Ubiquitinated CCNB1 proteins were immunoprecipitated with anti-Flag antibody-conjugated sepharose (Sigma Aldrich) and eluted with the Flag peptide. The purified ubiquitinated CCNB1 proteins were incubated with GST or GST-USP22 proteins in deubiquitination buffer (50 mm Tris-HCl, pH 8.0; 50 mm NaCl; 1 mm EDTA; 10 mm dithiothreitol and 5% glycerol) at 37 °C for 2 h. CCNB1 ubiquitination was detected by western blotting with anti-HA antibodies.

### *In vitro* kinase assay

GST-fusion proteins of USP22 and USP22/AA mutant, and the GST controls, were purified as reported [20]. Flag-CDK1/AF plasmids were transfected into HCT116 cells and the Flag-CDK1 protein in the lysate of transfected cells were purified by co-IP using anti-Flag antibody-conjugated sepherose and then incubated with GST-fusion proteins of USP22 or USP22/AA mutant, or the GST controls (10 μg each) in the *in vitro* kinase assay buffer (10 mm HEPES, pH 7.5; 50 mm Glycerophosphate; 50 mm NaCl; 10 mm MgCl_2_; 10 mm MnCl_2_; 5 μm ATP and 1 mm dithiothreitol) for 1 h at 37 °C. The levels of USP22 phosphorylation were detected by western blotting using anti-phospho-S/T antibodies and supersensitive enhanced chemiluminescence.

### Cell cycle analysis

Cells were seeded in a six-well dish at 1×10^6^ cells per well 1–2 days prior to analysis. The cells were collected and fixed in pre-cooled ethanol and incubated at −20 °C overnight. Cells were treated with 100 μg/ml RNAse in PBS, washed and stained with 5 μg ml^−1^ of propidium iodide. After washed with ice-cold PBS twice, cells were analyzed by flow cytometry and a Flowjo software (Portland, OR, USA).

### RNA extraction and real-time PCR analysis of gene expression

Total RNA was extracted using Trizol reagent (Invitrogen, San Diego, CA, USA). Quantitative real-time reverse transcriptase–PCR was performed using SYBR-Green quantitative PCR master mix (Clontech, San Diego, CA, USA). The *β-actin* gene was used as a reference for sample normalization. Primers for mouse or human genes including *β-actin* *, usp22, ccnb1, ccna, ccne, cdc20 and apc8* were purchased from RealTime Primers (Elkins Park, PA, USA). Standard amplification protocol was used according to the manufacturer’s instructions.

### Cell proliferation assay

*In vitro* cell proliferation was measured by using the colorimetric WST-1 assay as previously described (Awasthi *et al.*, 2009). Briefly, 4 000 cells were seeded in a 96-well plate with Dulbecco's Modified Eagle's Medium containing 10% fetal bovine serum. Every 24 h, 10 μl of WST-1 reagent was added to each well followed by incubation for 2 h. The absorbance at 450 nm was measured using a microplate reader.

### Soft agar colony formation assay

Cells were suspended at a low density (0.75×10^4^ cells) in 3 ml of culture medium containing 0.3% agar (USB Corporation, Cleveland, OH, USA) and seeded onto a base layer of 3 ml of 0.6% agar in 60-mm tissue culture dishes. After 3–4 weeks, colonies were stained, photographed and scored.

### Nude xenograft mice

We used a murine xenograft colon cancer model as described [48–50]. Briefly 1×10^6^ cells were injected subcutaneously into nude mice. The tumor volumes were measured using a caliper every other day. At day 22, all mice were euthanized and their tumors were isolated.

### Immunohistochemical analysis of USP22 and CCNB1 expression in human colon cancers

Micro tissue array slides that carry the paraffin-fixed human tumor tissues were purchased from Biomax (Rockville, MD, USA). A standard immunohistochemistry procedure was used for the analysis as described [20]. Briefly, after de-waxing with xylene followed by antigen retrieval, tissue sections were blocked by incubating them with 5% normal donkey serum. The slides were then incubated with primary antibodies against USP22 (Sigma, St Louis, MO, USA, 1:70 dilution) or CCNB1 (Cell Signaling, Cambridge, MA, USA, 1:70 dilution) overnight at 4 °C. The slides were then washed with PBST five times and incubated with biotinylated secondary antibodies (Vector Laboratories, Peterborough, UK, 1:400) followed by incubation with horseradish peroxidase-streptavidin. Horseradish peroxidase activity was detected with the Dab Substrate Kit (Vector Laboratories). Tissues were scored in a double-blinded manner. The expression levels of both USP22 and CCNB1 were scored by the following criteria: 0, No specific staining; 1; less than 25% of cells with strong staining or with less than 50% cells with weak staining; 2, less than 50% cells with strong staining or more than 50% cells with weak staining and 3, more than 50% cells with strong staining.

### Statistic analysis

We utilized a *χ*^2^-test to evaluate the relationship between USP22 and CCNB1 protein expression levels in human colon cancer tissues. Unpaired *t*-test was used to analyze the tumor volume means of xenograft nude mice. A two-tailed Student's *t-*test was used. *P*-values of <0.05 were considered to be statistically significant. Statistical analyses were performed using the Graphpad PRISM software (version 6, Graphpad Software Inc, La Jolla, CA, USA).
