# Supplementary material for: Ubiquitin-specific protease 22 is a deubiquitinase of CCNB1
Source: Cell Discov. 2015 Oct 13;1:15028–. doi: 10.1038/celldisc.2015.28 (PMC4809424; doi:10.1038/celldisc.2015.28)
Supplement: Supplementary Figures [file celldisc201528-s1.pdf]

Ubiquitin-specific Protease 22 is a Deubiquitinase of CCNB1

Zhenghong Lin [1], Can Tan [1], Quan Qiu [1], Sinyi Kong [1], Fang Zhao [1], Heeyoung Yang [1], Zhaojian Liu [1], Jinping Li [1], Qingfei Kong [1], Beixue Gao [1], Terry Barrett [2], Guang-Yu Yang [1], Jianing Zhang [3] and Deyu Fang [1, 3*]

Supplemental Figures 1-7 and legends:

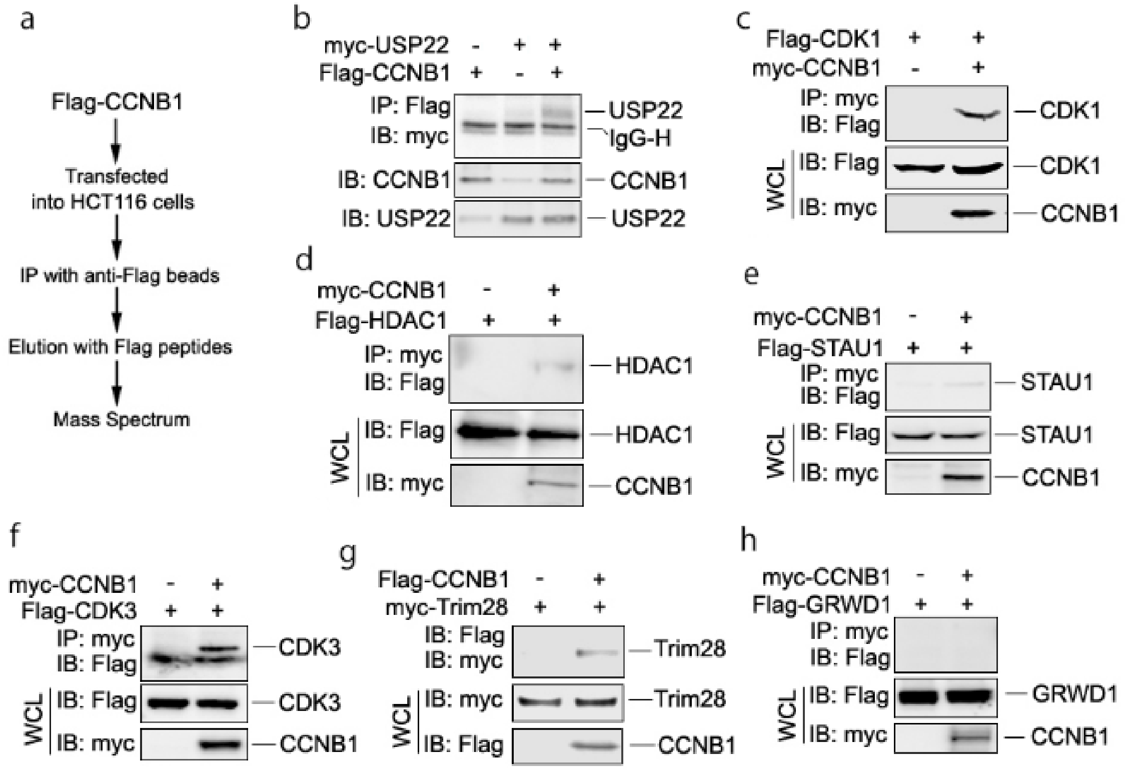

Figure S1. Isolation and validation of CCNB1-interacting proteins. (a) Diagram of the purification scheme for CCNB1-interacting proteins. See the Experimental Procedures for details. **(b)** myc-USP22, **(c)** Flag-CDK1, **(d)** Flag-HDAC1, **(e)** Flag-STAU1, **(f)** Flag-CDK3, **(g)** myc-Trim28, **(h)** Flag-GRWD1 and myc- or Flag-CCNB1 expression plasmids were co-transfected into HCT116 cells. The interactions of CCNB1 with each indicated protein in the transfected cells were determined by co-immunoprecipitation with anti-myc or anti-Flag and western blotting with each indicated antibody (top panels). The expression levels of CCNB1 and its co-transfected proteins in the whole cell lysates were analyzed by western blotting (middle and bottom panels).

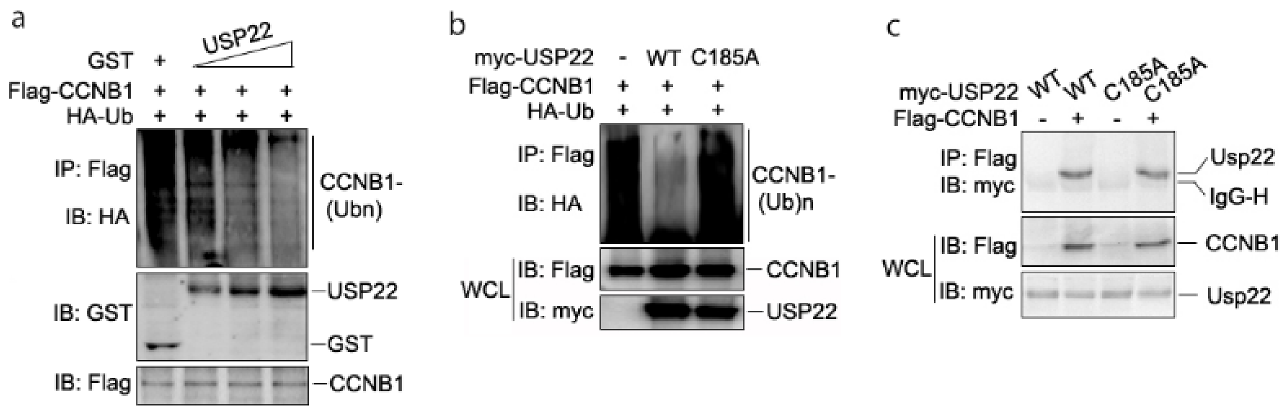

Figure S2. USP22 suppresses CCNB1 ubiquitination. (a) Deubiquitination of CCNB1 by USP22 in vitro. Ubiquitinated CCNB1 was purified from HCT116 cells transiently transfected with Flag-CCNB1 and HA-Ub expression plasmids, incubated with GST or with GST-USP22 fusion proteins at 37°C for 2 hours and then subjected to SDS-PAGE analysis. CCNB1 ubiquitination levels were determined by western blotting with anti-HA antibody (top panel). The expression levels of CCNB1 in the whole cell lysates and GST or GST-USP22 fusion protein were determined by western blotting with the indicated antibody. **(b)** Flag-CCNB1 and HA-Ub expression plasmids were co-transfected with vector, USP22 or its C185A mutant. CCNB1 ubiquitination was determined by immunoprecipitation (IP) of CCNB1 with anti-Flag antibodies and immunoblotting (IB) with anti-HA antibody (top panel). The expression levels of CCNB1 (middle panel) and USP22 (bottom panel) were confirmed by immunoblotting. **(c)** HCT116 cells were transfected with indicated plasmids and the interaction between CCNB1 and wild-type USP22 or its deubiquitinase catalytically inactive mutant was analyzed as in Figure S1B.

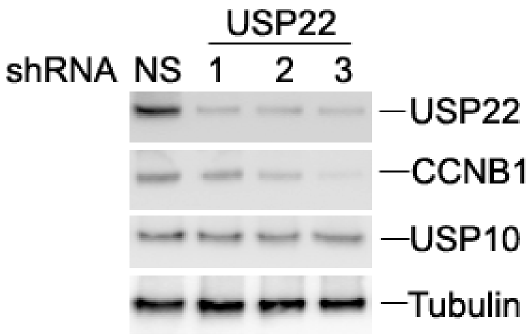

Figure S3. Knockdown of USP22 downregulates CCNB1 protein level. Three different shRNAs against USP22 were separately transfected into 293T cells using non-specific shRNA as control. USP22, USP10, and CCNB1 protein level was examined by western blotting with corresponding antibody.

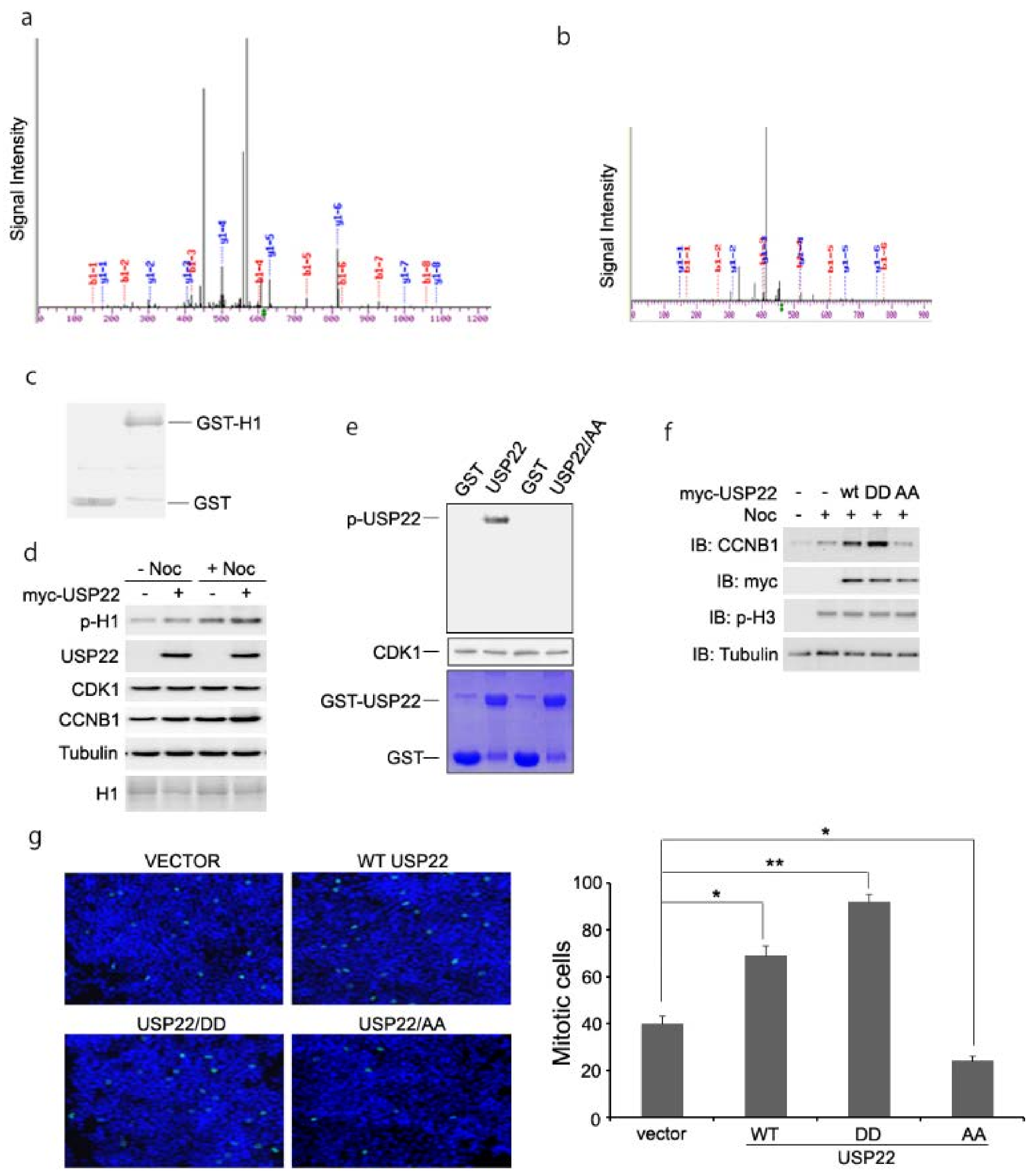

Figure S4. CDK1 phosphorylates USP22. (a & b) Proteomic analysis of USP22 phosphorylation. CDK1/AF was cotransfected with Flag-USP22 into HCT116 cells. USP22 proteins were immunoprecipitated, subjected to SDS-PAGE and then analyzed by mass spectrometry. The spectra of peptides carrying phosphorylated S237 (a) and T147 (b) are shown. **(c)** Histone H1 cDNA was cloned into

pGEX-6P1 vector and its expression in BL21 bacteria was tested by staining (top panel). HCT116 cells stably expressing USP22 or vector were treated without or with nocodazole for 12 hours. CDK1/CyclinB1 complexes were immunoprecipitated and an in vitro kinase assay was performed by using Histone H1 as substrate (bottom panel). **(d)** HCT116 cells were transfected with Flag-tagged CDK1/AF mutant plasmids. 48 hours later, CDK1/AF protein was immunoprecipitated and purified by anti-Flag antibody. In vitro kinase assay was performed by co-incubation of purified CDK1 and GST or GST-USP22 fusion protein in a buffer as depicted in materials and methods. Phosphorylated USP22 was detected by phosphor-serine/threnine antibody. **(e)** HCT116 cells expressing wt, phospho-mimetic USP22 (DD) or phosphor-inactive form of USP22 (AA) were treated with nocodazole as in (c). CCNB1 or USP22 expression was detected by indicated antibodies. Tubulin was used as loading control. **(f)** HCT116 cells expressing vector, wt USP22, phospho-mimetic USP22 (DD) or phosphor-inactive form of USP22 (AA) were subjected to immunofluorescence microscopy analysis to show the average numbers of cells that are mitotic by pH3 staining per 10X microscopy field (left panel). Mitotic cells from three independent experiments were counted and shown in right panel. * means significant difference, $p<0.05$; **, $p<0.01$.

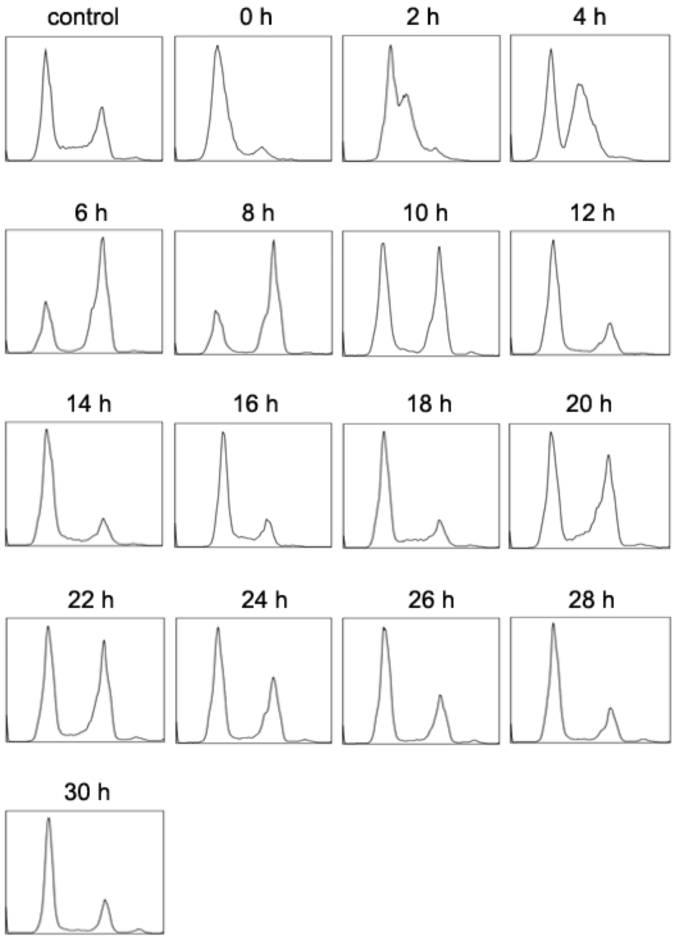

Figure S5. FACS analysis of the cell cycle. HCT116 cells were released from a double-thymidine block, cells were collected every two hours after release, and FACS analysis was performed.

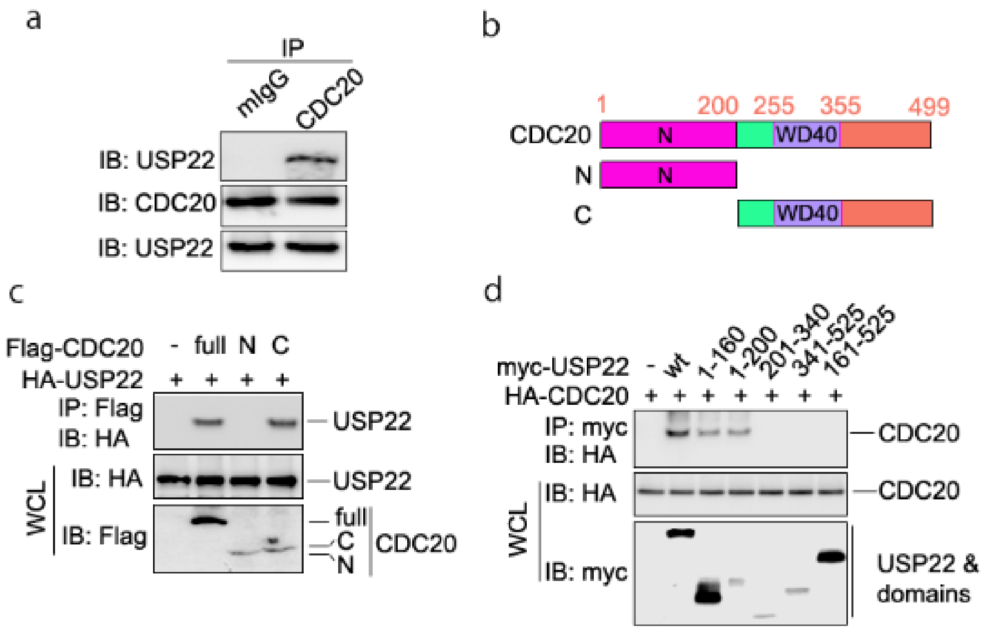

Figure S6. USP22 interacts with CDC20. (a) HCT116 cells were lysed, and the interaction between endogenous CDC20 and USP22 was determined by immunoprecipitation of CDC20 using normal mouse IgG as control and immunoblotting with anti-USP22 antibody. **(b)** Domain structures of CDC20 and its truncated mutants. **(c)** USP22 expression plasmids were cotransfected with CDC20 or each of the truncated mutants shown in (B) into 293T cells. The interaction between CDC20 or its mutants and USP22 was examined. **(d)** CDC20 expression plasmids were cotransfected with USP22 or each of the truncated mutants into 293T cells. The interaction between USP22 or its mutants and CDC20 was examined.

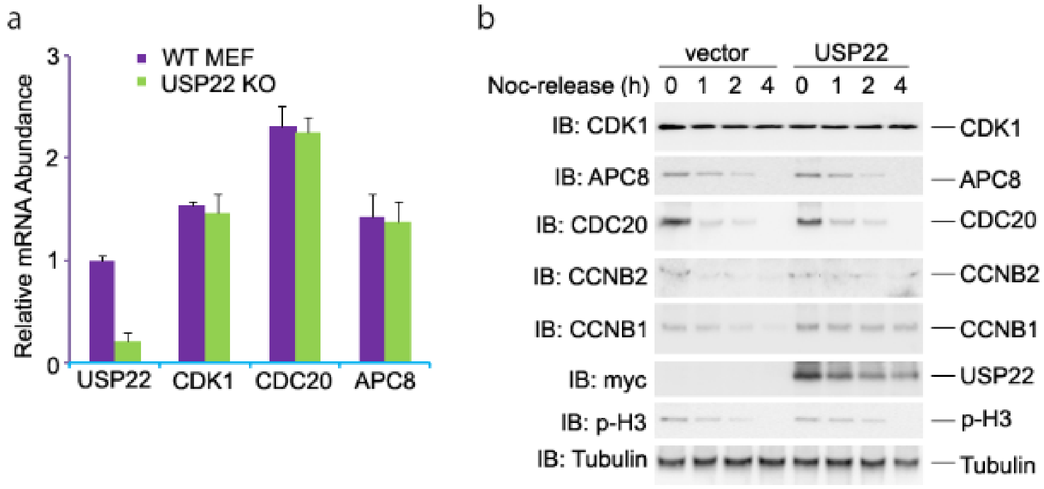

Figure S7. USP22 specifically stabilizes CCNB1. (a) CDK1, CDC20, and APC8 mRNA were not affected by USP22 deletion. The mRNA levels of USP22, CDK1, CDC20, and APC8 in MEF cells were analyzed by real-time PCR. Error bars represent data from three independent experiments. **(b)** USP22 stabilizes CCNB1 but not APC8, CDC20, CDK1 or CCNB2. HCT116 cells expressing USP22 or vector were treated with nocodazole for 12 hours. Mitotic cells were collected by shake-off method and then released into fresh medium for indicated times. USP22, CCNB1, APC8, CDC20, CDK1, CCNB2 or Phospho-H3 level was detected by corresponding antibody. Tubulin was used as loading control.
